# Pontine Tegmental Cap Dysplasia Presenting With Global Developmental Delay and Vestibulocochlear Nerve Aplasia: A Case Report

**DOI:** 10.1002/ccr3.73408

**Published:** 2026-08-23

**Authors:** Khawar Bilal, Muhammad Owais Rao, Syed Muhammed Salman Hassan, Arnella Husenaj Tagani, Naila Nadeem, Sarim Hassan Shahab, Fazal Habib

**Affiliations:** ^1^ Aga Khan University Hospital Karachi Pakistan; ^2^ Nishtar Medical University Multan Pakistan; ^3^ University of Prishtina Pristina Kosovo; ^4^ Sher‐E‐Bangla Medical College Barisal Bangladesh

**Keywords:** global developmental delay, magnetic resonance imaging, pontine tegmental cap dysplasia, sensorineural hearing loss, vestibulocochlear nerve aplasia

## Abstract

PTCD should be considered in children with developmental delay and sensorineural hearing loss. MRI is crucial for identifying the characteristic dorsal “tegmental cap” and associated hindbrain anomalies. Vestibulocochlear nerve aplasia may explain severe hearing impairment in PTCD. Early diagnosis enables timely multidisciplinary rehabilitation and audiological intervention.

## 1 Introduction

Global developmental delay is a common pediatric concern with diverse etiologies, including structural brain abnormalities, genetic conditions, and perinatal insults. Neuroimaging plays a crucial role in identifying underlying causes.

Pontine tegmental cap dysplasia (PTCD) is a rare brainstem malformation resulting from abnormal development of pontocerebellar pathways. It is characterized by ectopic transverse fibers in the dorsal pons forming a “tegmental cap” on MRI. Clinically, patients often present with developmental delay and cranial nerve dysfunction. Due to its rarity, the diagnosis may be overlooked without careful neuroimaging evaluation and correlation with previously reported imaging findings [1, 2, 3].

## 2 Case Presentation

A 20‐month‐old female presented with delayed walking and concerns regarding hearing impairment. Developmental assessment revealed delays across gross motor, fine motor, and speech domains. She was not walking independently and had limited expressive speech.

Audiological evaluation using brainstem evoked response audiometry (BERA) confirmed bilateral sensorineural hearing loss.

Antenatal ultrasound had suggested hydrocephalus. The patient was born at term via spontaneous vaginal delivery with normal Apgar scores. On the first day of life, she developed poor feeding and lethargy, requiring neonatal unit admission. She required incubator support for 1 week. Due to a poor sucking reflex, nasogastric tube feeding was initiated at 6 months of age to ensure adequate nutritional support. Following approximately 3 months of enteral nutritional support, serial clinical assessments demonstrated improvement in feeding ability, allowing successful transition to bottle feeding and subsequent removal of the nasogastric tube. Developmental delays became increasingly evident during infancy. At the most recent follow‐up at 20 months of age, the patient is feeding orally without difficulty and no longer requires enteral nutritional support. She tolerates bottle‐fed liquids and consumes semisolid and solid foods, including age‐appropriate foods, with a good appetite. Her nutritional status is satisfactory, with both weight and height tracking at approximately the 40th percentile for age, indicating appropriate growth trajectory despite early feeding difficulties.

## 3 Investigations

### 3.1 Audiology

BERA: Bilateral sensorineural hearing loss.

### 3.2 MRI Brain and Internal Auditory Canals

**FIGURE 1:**
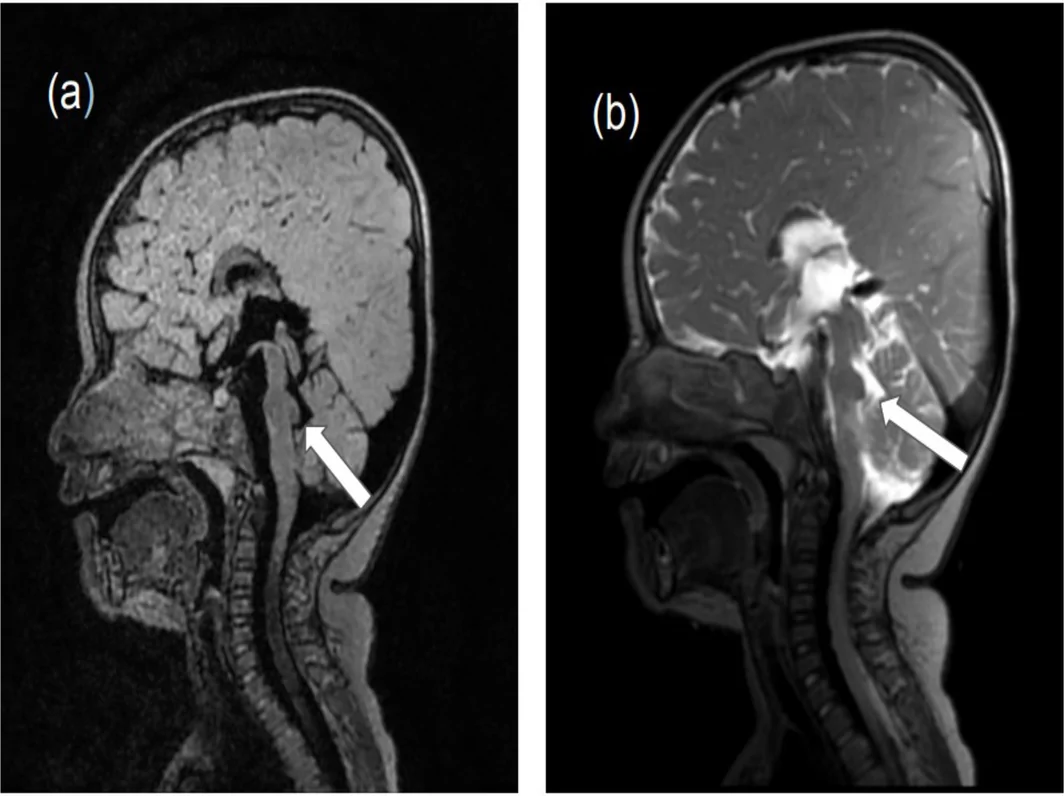
Sagittal Flair (a) and sagittal T2 weighted (b) images show soft tissue arising from pontine tegmentum (white arrow) representing ectopic dorsal transverse pontine fibers.

**FIGURE 2:**
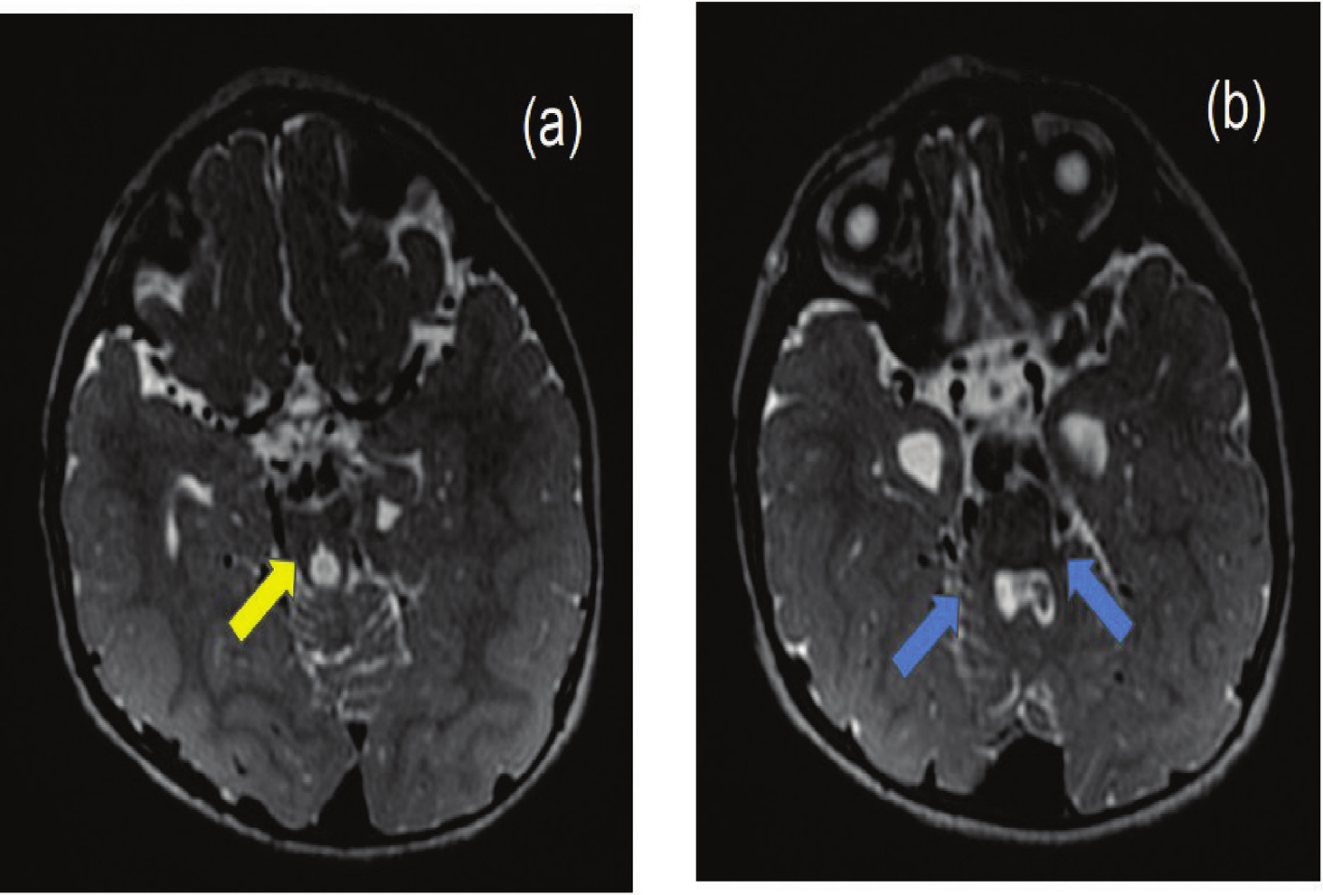
Axial T2‐weighted image (a) shows lateralized course of superior cerebellar peduncles giving “Molar tooth” appearance (Yellow arrow), and (b) shows hypoplastic bilateral middle cerebellar peduncles (blue arrows).

**FIGURE 3:**
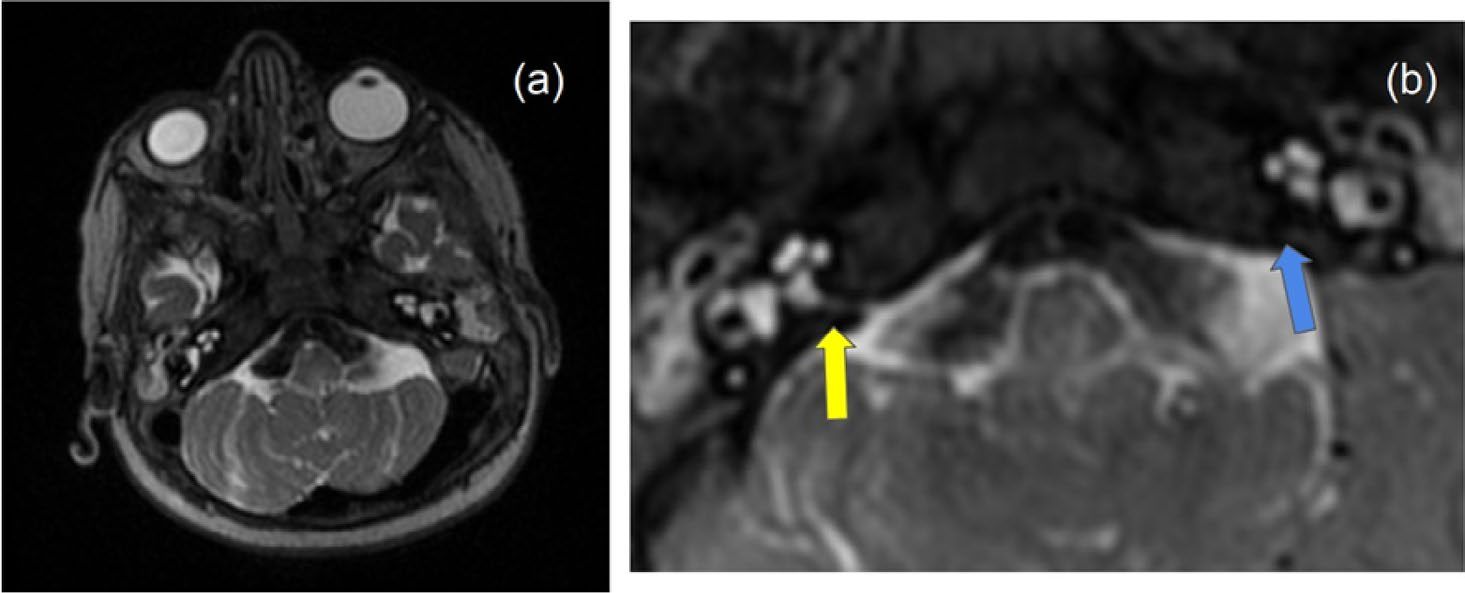
Axial T2‐weighted image (a) shows normal formation of bilateral internal auditory apparatus, however axial T2 weighted magnified images (b) shows dysplastic right (Yellow arrow) and aplastic left (Blue arrow) internal auditory canals, suggesting vestibulocochlear aplasia.

MRI demonstrated features consistent with PTCD, including dorsal pontine “tegmental cap,” ventral pontine hypoplasia, and abnormal cerebellar peduncle morphology. High‐resolution imaging of the internal auditory canals demonstrated bilateral absence of the vestibulocochlear nerves, with preserved facial nerves (Figures 1, 2, 3).

## 4 Diagnosis

Pontine tegmental cap dysplasia with bilateral vestibulocochlear nerve aplasia, associated with global developmental delay.

## 5 Differential Diagnosis

Radiological Differential Diagnosis: The radiological differentials in our case included Horizontal Gaze Palsy with Progressive Scoliosis (HGPPS), Joubert Syndrome, and Dandy–Walker malformation. In our case, the presence of a characteristic dorsal pontine “tegmental cap,” ventral pontine hypoplasia, abnormal cerebellar peduncles, and bilateral vestibulocochlear nerve aplasia was the defining MRI feature favoring PTCD. A molar tooth appearance was also identified on MRI; while this sign is classically associated with Joubert syndrome, it may occasionally be seen in PTCD due to overlapping hindbrain malformation features. The critical radiological differentiators from Joubert syndrome in our patient were the presence of the dorsal exophytic tegmental cap, bilateral vestibulocochlear nerve aplasia, and the absence of cerebellar vermis hypoplasia or supratentorial anomalies characteristic of Joubert syndrome. Unlike HGPPS, no split‐pons or butterfly configuration was identified on MRI. Dandy–Walker malformation was excluded by the absence of cystic fourth ventricular dilation and posterior fossa enlargement. Clinical Differential Diagnosis: Clinically, the differential diagnosis encompassed Möbius Syndrome, Duane Retraction Syndrome, and other causes of global developmental delay with sensorineural hearing loss. Möbius syndrome was considered given the history of poor neonatal feeding and developmental delay; however, it primarily manifests as congenital facial diplegia and abducens nerve palsy without the characteristic pontine tegmental cap or extensive pontocerebellar anomalies seen in this patient. Duane Retraction Syndrome was excluded clinically as it presents with restricted horizontal gaze due to aberrant innervation of the lateral rectus muscle and lacks the broader brainstem and cranial nerve involvement observed in our case. CHARGE syndrome, which can present with coloboma, choanal atresia, genital anomalies, ear anomalies, and sensorineural hearing loss, was also considered; however, the absence of these associated features and the presence of the characteristic pontine tegmental cap on MRI argued against this diagnosis. The combination of global developmental delay, bilateral sensorineural hearing loss secondary to vestibulocochlear nerve aplasia, and the characteristic neuroimaging constellation collectively supported a diagnosis of PTCD.

## 6 Discussion

Pontine tegmental cap dysplasia (PTCD) is a rare congenital malformation of the brainstem attributed to abnormal axonal guidance and disrupted pontocerebellar fiber decussation during embryogenesis. It is characterized by a distinctive dorsal “tegmental cap” formed by ectopic transverse pontine fibers projecting into the fourth ventricle, along with hypoplasia of the ventral pons and abnormal cerebellar peduncles [1, 2].

MRI is the diagnostic modality of choice, demonstrating the characteristic constellation of findings. Advanced techniques such as diffusion tensor imaging (DTI) can further illustrate disruption of normal pontocerebellar tract decussation, supporting the theory of axonal misguidance [1, 2].

The differential diagnosis includes Joubert syndrome and Dandy–Walker malformation. Although the molar tooth sign is classically associated with Joubert syndrome, it may occasionally be observed in PTCD due to overlapping hindbrain malformation features, as was present in this case [3, 4]. The critical distinguishing features in our patient were the dorsal exophytic pontine tegmental cap, bilateral vestibulocochlear nerve aplasia, and the absence of cerebellar vermis hypoplasia or the characteristic supratentorial anomalies of Joubert syndrome, which collectively favored a diagnosis of PTCD. Dandy–Walker malformation presents with cystic dilation of the fourth ventricle and enlargement of the posterior fossa, features not seen in PTCD [2, 5].

Clinically, PTCD presents with developmental delay, hypotonia, and cranial nerve dysfunction. Reported cranial neuropathies include involvement of facial, trigeminal, and vestibulocochlear nerves, consistent with widespread brainstem and cranial nerve developmental abnormalities [1, 2, 5].

In this case, bilateral internal auditory canal abnormalities with absence of the vestibulocochlear nerves explain the severe sensorineural hearing loss confirmed by BERA. High‐resolution heavily T2‐weighted sequences (such as CISS/FIESTA) are critical for evaluating cranial nerve integrity within the cerebellopontine angle and internal auditory canals [6, 7].

Although not widely reported as a defining feature of PTCD, vestibulocochlear nerve aplasia in this case suggests a more extensive developmental disruption involving cranial nerve formation.

Antenatal hydrocephalus in this patient may reflect early impairment of cerebrospinal fluid dynamics related to abnormal hindbrain development rather than isolated obstructive pathology. Similar antenatal findings have been described in posterior fossa malformations [8].

From a developmental perspective, PTCD is thought to result from disruption of early axonal guidance pathways governing midline crossing and pontocerebellar tract formation, with overlap in mechanisms described in other hindbrain malformations [1, 2].

Management is supportive and requires a multidisciplinary approach. In this case, several specific interventions were initiated or considered following diagnosis. Audiologic rehabilitation was discussed following confirmation of bilateral vestibulocochlear nerve deficiency, characterized by aplasia of the left internal auditory canal and severe hypoplasia of the right internal auditory canal. Given the absence or marked deficiency of the cochlear nerves, conventional cochlear implantation was deemed unsuitable. An auditory brainstem implant (ABI) was considered as a potential alternative; however, this procedure is currently unavailable in our country and therefore could not be offered. The patient remains nonverbal at 20 months and primarily communicates through gestures and nonverbal cues; sign language support and augmentative communication strategies have been recommended to the family. Regarding motor development, the patient is receiving regular physiotherapy at our institution, and her parents have been instructed in home‐based physiotherapy exercises performed routinely to support gross motor development. She is currently able to roll over and sit independently but has not yet achieved independent standing or walking, with bilateral lower‐limb weakness remaining evident. Multidisciplinary follow‐up continues with developmental monitoring, nutritional surveillance, and communication support. No recurrent infections or other significant medical complications have been reported. Early identification of hearing loss and cranial nerve dysfunction is particularly important in PTCD, as timely intervention may optimize developmental outcomes.

## 7 Conclusion

Pontine tegmental cap dysplasia is a rare hindbrain malformation that can present with global developmental delay, cranial nerve abnormalities, and sensorineural hearing loss. MRI is essential for diagnosis, demonstrating the characteristic tegmental cap appearance and associated pontocerebellar anomalies. Associated vestibulocochlear nerve aplasia, as seen in this patient, explains the profound sensorineural hearing loss and should be specifically evaluated using high‐resolution MRI sequences. Although the molar tooth sign may occasionally be observed in PTCD due to overlapping hindbrain features, differentiation from Joubert syndrome relies on the presence of the dorsal tegmental cap and VCN aplasia. Early recognition enables appropriate multidisciplinary management. When conventional cochlear implantation is not feasible due to cochlear nerve absence, auditory brainstem implantation should be considered where available. Physiotherapy, communication support, and developmental surveillance are cornerstones of management. This case underscores the importance of comprehensive neuroimaging and a coordinated multidisciplinary approach in children presenting with developmental delay and sensorineural hearing loss.

## Author Contributions

**Syed Muhammed Salman Hassan:** writing – review and editing, writing – original draft. **Khawar Bilal:** conceptualization, writing – original draft, writing – review and editing, project administration. **Naila Nadeem:** writing – review and editing, writing – original draft. **Fazal Habib:** writing – review and editing. **Sarim Hassan Shahab:** writing – review and editing. **Arnella Husenaj Tagani:** writing – review and editing, writing – original draft. **Muhammad Owais Rao:** writing – review and editing, writing – original draft.

## Funding

The authors have nothing to report.

## Ethics Statement

The authors have nothing to report.

## Consent

Written informed consent was obtained from the patient's parents for publication of this case report and accompanying images.

## Conflicts of Interest

The authors declare no conflicts of interest.

## Data Availability

Data sharing not applicable to this article as no datasets were generated or analysed during the current study.
